# Noise Robustness of Transcript-Based Estimators for Properties of Interactions

**DOI:** 10.3390/e27101067

**Published:** 2025-10-14

**Authors:** Manuel Adams, Klaus Lehnertz

**Affiliations:** 1 Department of Epileptology, University Hospital Bonn, Venusberg-Campus 1, 53127 Bonn, Germany; klaus.lehnertz@ukbonn.de; 2 Helmholtz Institute for Radiation and Nuclear Physics, University of Bonn, Nussallee 14-16, 53115 Bonn, Germany; 3 Interdisciplinary Center for Complex Systems, University of Bonn, Brühler Straße 7, 53175 Bonn, Germany

**Keywords:** transcripts, ordinal patterns, colored noise, interactions, synchronization, directionality, complexity

## Abstract

We investigate the robustness of transcript-based estimators for properties of interactions against various types of noise, ranging from colored noise to isospectral noise. We observe that all estimators are sensitive to symmetric and asymmetric contamination at signal-to-noise ratios that are orders of magnitude higher than those typically encountered in real-world applications. While different coupling regimes can still be distinguished and characterized sufficiently well, the strong impact of noise on the estimator for the direction of interaction can lead to severe misinterpretations of the underlying coupling structure.

## 1. Introduction

Robustly characterizing properties of couplings—such as directionality, strength, and complexity—between interacting systems is of great interest in diverse scientific fields ranging from physics and chemistry via earth sciences to neuroscience [1,2,3,4,5,6,7,8,9]. An improved understanding of the nature of such couplings would enable eventually the full reconstruction of the functional relationship between the systems under investigation. Quite often a direct (i.e., perturbation-based) access to coupling is not possible, and in this case, one usually resorts to linear and nonlinear time-series-analysis techniques to quantify properties of couplings from pairs of time series of appropriate system observables. Since couplings can manifest themselves in various aspects of the dynamics, a multitude of analysis techniques, originating from statistics, synchronization theory, nonlinear dynamics, information theory, statistical physics, and from the theory of stochastic processes, have been developed yielding promising results. Recent advances in the field of symbolic dynamics [10,11,12] have provided us with ordinal-pattern- and transcript-based analysis techniques [13,14,15,16,17,18,19,20,21] that combine information-theoretic estimators with permutation-theoretic concepts to enable the characterization of dynamical systems and of aspects of interactions between them using a singular concept [22,23,24,25,26,27].

A frequently mentioned advantage of the aforementioned time-series-analysis techniques is their relatively high robustness against observational or measurement noise (see [28,29] and references therein). It is, however, not clear whether the robustness also holds for contamination with other types of noise (such as colored [30,31,32,33] and isospectral noise [34]), particularly if techniques are employed to characterize properties of couplings.

In this paper, we investigate the robustness of transcript-based estimators for the direction, strength, and complexity of interactions [16,35,36,37,38] under the impact of nondeterministic linear noise contributions with a ${\left(1/f\right)}^{\beta }$ spectrum as well as of isospectral (or in-band) noise. We mimic conditions that are found in the analysis of real-world data and use coupled chaotic maps and oscillators, whose dynamics we superimpose with the aforementioned types of noise in a symmetric and asymmetric fashion.

## 2. Methods

### 2.1. Ordinal Patterns, Transcripts, and Order Classes

A time series of length *N* is divided into delay vectors of size *d* using a delay embedding [13,39,40] with delay *m*, leading to a series of L=N−(d−1)m delay vectors. Ordinal patterns or symbols can then be derived by rank-ordering the amplitude values in the corresponding delay vectors from lowest to highest, thus creating values between 0 and d−1. For example, with d=4 and m=1, for the delay vector q=(1.9,2.3,−0.5,0.5), the corresponding ordinal pattern is $\theta =\left[\theta_{0},\theta_{1},\theta_{2},\theta_{3}\right]=\left[2,3,0,1\right]$. If a vector contains the same value multiple times, we choose to order the entries from left to right, i.e., the unit symbol I=[0,1,2,…,d−1] relates to both a vector containing constant or monotonously increasing values.

Through the framework of permutation theory, we are able to characterize the relationship between two ordinal patterns. In the following, $S_{d}$ denotes the set of ordinal patterns of length *d*. Given two ordinal patterns $\mu ,\nu \in S_{d}$, the transcript $\tau \in S_{d}$ with elements $\left[\tau_{0},\tau_{1},\ldots ,\tau_{d-1}\right]$ from $\mu =[\mu_{0},\mu_{1},\ldots ,\mu_{d-1}]$ to $\nu =[\nu_{0},\nu_{1},\ldots ,\nu_{d-1}]$, where each element of μ,ν, and τ is an element of the set {0,1,…,d−1}, is defined as [41]

(1) τ∘μ=ν,

with the two ordinal patterns acting onto each other via the composition

(2) $$\tau \circ \mu =\left[\mu_{\tau_{0}},\mu_{\tau_{1}},\ldots ,\mu_{\tau_{d-1}}\right]=\left[\nu_{0},\nu_{1},\ldots ,\nu_{d-1}\right].$$

The inverse element of this group can be found by the following considerations (we will omit the composition symbol ∘ in the following). Since for the inverse element, we know that

(3) $$\tau^{-1}\tau =\left[\tau_{{\left(\tau^{-1}\right)}_{0}},\tau_{{\left(\tau^{-1}\right)}_{1}},\ldots ,\tau_{{\left(\tau^{-1}\right)}_{d-1}}\right]\overset{!}{=}I,$$

it follows directly that

(4) $$\tau_{{\left(\tau^{-1}\right)}_{0}}<\tau_{{\left(\tau^{-1}\right)}_{1}}<\ldots <\tau_{{\left(\tau^{-1}\right)}_{d-1}}.$$

With the above definitions, given two series of ordinal patterns μ and ν, a transcript can then be calculated via $\tau =\nu \mu^{-1}$.

The set $S_{d}$ can be partitioned into different non-overlapping subsets of symbols according to their permutation order *n*, with

(5) $$n=\underset{n\in N_{>0}}{\arg\;\in }\left(\tau^{n}=I\right).$$

In the following, these subsets will be denoted as order classes $C_{n}$. Thus, the order *n* of a transcript τ describes the minimum number of non-trivial operations needed to obtain the unit symbol.

### 2.2. Transcript-Based Estimators for Direction, Strength, and Complexity of an Interaction

Given two (supposedly coupled) dynamical systems *X* and *Y* with corresponding ordinal pattern series $\hat{\alpha }$ and $\hat{\beta }$ and their respective ordinal patterns ${\left\{{\hat{\alpha }}_{i}\right\}}_{i=0}^{L-1}$ and ${\left\{{\hat{\beta }}_{i}\right\}}_{i=0}^{L-1}$, we define the transcript series ${\hat{\tau }}^{\left(\hat{\alpha },\hat{\beta }\right)}$ with ${\hat{\tau }}_{i}^{\left(\hat{\alpha },\hat{\beta }\right)}{\hat{\alpha }}_{i}={\hat{\beta }}_{i}$, where ${\hat{\alpha }}_{i},{\hat{\beta }}_{i},{\hat{\tau }}_{i}^{\left(\cdot ,\cdot \right)}\in S_{d}$. As an estimator for the direction of an interaction [37], we here consider the mutual information of transcripts which, for three arbitrary ordinal pattern series $\hat{\alpha }$, $\hat{\beta }$ and $\hat{\gamma }$ is defined as

(6) $$I\left({\hat{\tau }}^{\left(\hat{\gamma },\hat{\alpha }\right)},{\hat{\tau }}^{\left(\hat{\beta },\hat{\alpha }\right)}\right)=H\left({\hat{\tau }}^{\left(\hat{\gamma },\hat{\alpha }\right)}\right)-H\left({\hat{\tau }}^{\left(\hat{\gamma },\hat{\alpha }\right)}|{\hat{\tau }}^{\left(\hat{\beta },\hat{\alpha }\right)}\right),$$

with the entropy H(·), and ${\hat{\tau }}^{\left(\hat{\alpha },\hat{\beta }\right)}$ denoting, e.g., the transcript series between the ordinal patterns of series $\hat{\alpha }$ and $\hat{\beta }$. When employing this measure to characterize an interaction between two systems, we use an approach similar to the one used for the definition of the symbolic transfer entropy [14,42]. We first define

(7) $$\begin{array}{cc} {\hat{\tau }}^{\left(\hat{\alpha },{\hat{\alpha }}^{*}\right)}\hat{\alpha } & ={\hat{\alpha }}^{*}\;,\\ {\hat{\tau }}^{\left(\hat{\beta },{\hat{\beta }}^{*}\right)}\hat{\beta } & ={\hat{\beta }}^{*}\;, \end{array}\;\begin{array}{cc} {\hat{\alpha }}_{i}^{*} & ={\hat{\alpha }}_{i+\Lambda }\;,\\ {\hat{\beta }}_{i}^{*} & ={\hat{\beta }}_{i+\Lambda }\;, \end{array}$$

where ${\hat{\alpha }}^{*}$ denotes the ordinal pattern series $\hat{\alpha }$ shifted elementwise in time by a delay Λ (which is not to be confused with the embedding delay *m*). With Λ=1 and using Equation (7), the transcript-based directionality index can here be defined as

(8) $$T^{\left(\hat{\tau }\right)}=I\left({\hat{\tau }}^{\left(\hat{\beta },{\hat{\beta }}^{*}\right)},{\hat{\tau }}^{\left(\hat{\alpha },\hat{\beta }\right)}\right)-I\left({\hat{\tau }}^{\left(\hat{\alpha },{\hat{\alpha }}^{*}\right)},{\hat{\tau }}^{\left(\hat{\beta },\hat{\alpha }\right)}\right),$$

where $T^{\left(\hat{\tau }\right)}$ exploits the asymmetry of the mutual information of transcripts (Equation (6)) under the interchange of the series $\hat{\alpha }$ and $\hat{\beta }$. It can be seen that for a direction of interaction from system *X* to system *Y*, $T^{\left(\hat{\tau }\right)}$ takes on positive values while for a direction of interaction from *Y* to *X*, it becomes negative. $T^{\left(\hat{\tau }\right)}=0$ for uncoupled or bidirectionally coupled systems.

As an estimator for the strength of an interaction [35], the Jensen–Shannon (JS-) divergence compares the probability densities of the order classes of coupled systems with the probability densities that are expected for independent systems. We define the probability densities for transcripts as

(9) $$P\left(\tau \right)=\sum_{\mu ,\nu \in S_{d}:\nu \mu^{-1}=\tau }P^{J}\left(\mu ,\nu \right),$$

with $P^{J}$ denoting the joint probability of the pair (μ, ν) in ordinal pattern series $\hat{\alpha }$ and $\hat{\beta }$, and

(10) $$P^{\mathrm{ind}}\left(\tau \right)=\sum_{\mu ,\nu \in S_{d}:\nu \mu^{-1}=\tau }P_{\hat{\alpha }}\left(\mu \right)P_{\hat{\beta }}\left(\nu \right)$$

the probability density of transcriptions for independent pairs. $P_{\hat{\alpha }}\left(\mu \right)$ ($P_{\hat{\beta }}\left(\nu \right)$) here denotes the probability of ordinal pattern μ (ν) in the ordinal pattern series $\hat{\alpha }$ ($\hat{\beta }$). Similarly, one can derive the JS-divergence for order classes by restricting the sums over ordinal patterns to sums over order classes, thus altering the resulting probability densities. With

(11) $$P_{C_{n}}:=\sum_{\tau \in C_{n}}P\left(\tau \right),$$

(12) $$P_{C_{n}}^{\mathrm{ind}}:=\sum_{\tau \in C_{n}}P^{\mathrm{ind}}\left(\tau \right),$$

we obtain the Kullback–Leibler divergence for order classes [35]:

(13) $$E_{\mathrm{KL}}(P_{C}||P_{C}^{\mathrm{ind}})=\sum_{n}P_{C_{n}}\log_{2}\left(P_{C_{n}}/P_{C_{n}}^{\mathrm{ind}}\right).$$

With the mixing probability

(14) $$P_{C_{n}}^{M}:=\frac{1}{2}P_{C_{n}}+\frac{1}{2}P_{C_{n}}^{\mathrm{ind}},$$

the JS-divergence for order classes is defined as

(15) $$D_{\mathrm{JS}}^{C}(P_{C}||P_{C}^{\mathrm{ind}})=\frac{1}{2}\left(E_{\mathrm{KL}}(P_{C}||P_{C}^{M})+E_{\mathrm{KL}}(P_{C}^{\mathrm{ind}}||P_{C}^{M})\right).$$

$D_{\mathrm{JS}}^{C}\geq 0$, because $E_{\mathrm{KL}}\geq 0$. The upper bound of $D_{\mathrm{JS}}^{C}$ depends on the chosen embedding dimension and can be determined analytically [38].

Complexity C [16,36] of an interaction (or coupling) can be quantified by taking into account the entropies $H(\hat{\alpha })$ and $H(\hat{\beta })$ of the series and their relationships, characterized by the transcripts ${\hat{\tau }}^{\left(\hat{\alpha },\hat{\beta }\right)}$ between them:

(16) $$C\left(\hat{\alpha },\hat{\beta }\right)=\min\left[H\left(\hat{\alpha }\right),H\left(\hat{\beta }\right)\right]-\Delta \left(\hat{\alpha },\hat{\beta }\right),$$

where

(17) $$\Delta \left(\hat{\alpha },\hat{\beta }\right)=H\left(\hat{\alpha },\hat{\beta }\right)-H\left({\hat{\tau }}^{\left(\hat{\alpha },\hat{\beta }\right)}\right)$$

describes the information loss of the transcription process. Note that C is a symmetric measure under the interchange of series $\hat{\alpha }$ and $\hat{\beta }$ because it is a characteristic of the relationship between dynamical processes [16].

## 3. Model Systems

For our investigations, we considered coupled model systems with well-known properties, namely two unidirectionally coupled Hénon maps as well as two bidirectionally coupled Rössler oscillators.

The equations of motion for the first system read [43]

(18) $$\begin{array}{ccc} x_{1,n+1} & = & a-x_{1,n}^{2}+b_{1}y_{1,n},\\ y_{1,n+1} & = & x_{1,n},\\ x_{2,n+1} & = & a-\left(kx_{1,n}x_{2,n}+\left(1-k\right)x_{2,n}^{2}\right)+b_{2}y_{2,n},\\ y_{2,n+1} & = & x_{2,n}. \end{array}$$

We let a=1.4 and $b_{1}=b_{2}=0.3$ and varied the coupling strength *k* from 0 to 1 in steps of 0.01, with initial conditions picked randomly from the interval [0, 1]. After discarding transient data, we iterated the system over *T* time steps.

For the second system, the equations of motion read [35]

(19) $$\begin{array}{ccc} {\dot{x}}_{1} & = & -\left(\omega_{1}y_{1}+z_{1}\right)+k\left(x_{1}-x_{2}\right),\\ {\dot{y}}_{1} & = & \omega_{1}x_{1}+0.165y_{1},\\ {\dot{z}}_{1} & = & 0.2+z_{1}\left(x_{1}-10\right),\\ {\dot{x}}_{2} & = & -\left(\omega_{2}y_{2}+z_{2}\right)+k\left(x_{2}-x_{1}\right),\\ {\dot{y}}_{2} & = & \omega_{2}x_{2}+0.165y_{2},\\ {\dot{z}}_{2} & = & 0.2+z_{2}\left(x_{2}-10\right). \end{array}$$

With a slight mismatch in the eigenfrequencies ($\omega_{1}=0.99$ and $\omega_{2}=0.95$), we integrated the system using a Runge–Kutta integrator of order 5(4) of the Dormand–Prince class, choosing random initial conditions from the interval [−5, 5]. The integration step was δt=0.001. After discarding transient data, the system was integrated over *T* time steps and we created time series using a sampling interval Δt=0.01. The coupling strength *k* has been varied between 0 and 0.300 in steps of Δk=0.001.

For both systems, we investigated interactions between *x*-components of the respective subsystems, both for the noise-free and noise-contaminated cases. In addition to white noise (additive Gaussian δ-correlated noise), which is often used to mimic the influence of observational noise, here we examined the impact of nondeterministic linear noise contributions, which exhibit a ${\left(1/f\right)}^{\beta }$ spectrum with $\beta \in \left\{2,1,-1,-2\right\}$ [44] (in the following, we refer to these contributions as $f^{-2}$-, $f^{-1}$-, $f^{0}$-, $f^{1}$-, and $f^{2}$-noise, respectively). For the coupled Rössler oscillators, we also investigated the influence of isospectral noise. To this end, we generated surrogate time series [45] of the model time series from each subsystem using the iterative amplitude adjusted Fourier transform (IAAFT) method.

We considered symmetric (noise added to both subsystems) and asymmetric noise contamination (noise added to only one subsystem [46]), for both with a signal-to-noise ratio $\rho =\sigma_{\mathrm{signal}}^{2}/\sigma_{\mathrm{noise}}^{2}$. We generated time-series of length $T=2^{16}$ for the coupled Hénon maps and of $T=2^{17}$ for the coupled Rössler oscillators. For each value of the coupling strength *k*, the signal-to-noise ratio was varied from ρ=0.1 to ρ=1000. For each pair (k,ρ), we generated 20 different realizations of the noise that we added to the same realization of the model time series.

For the unidirectionally coupled Hénon maps, we chose an embedding delay m=1, based on a visual inspection of the return map of the uncoupled, noise-free *x*-component. We fixed the embedding dimension at d=4. For the bidirectionally coupled Rössler oscillators, we chose an embedding delay of m=150, derived from the first zero-crossing of the autocorrelation function of the *x*-component in the uncoupled, noise-free case. The embedding dimension is fixed at d=6.

In the following, we report mean values of the transcript-based estimators for direction, strength, and complexity of an interaction.

## 4. Results

**Figure 1:**
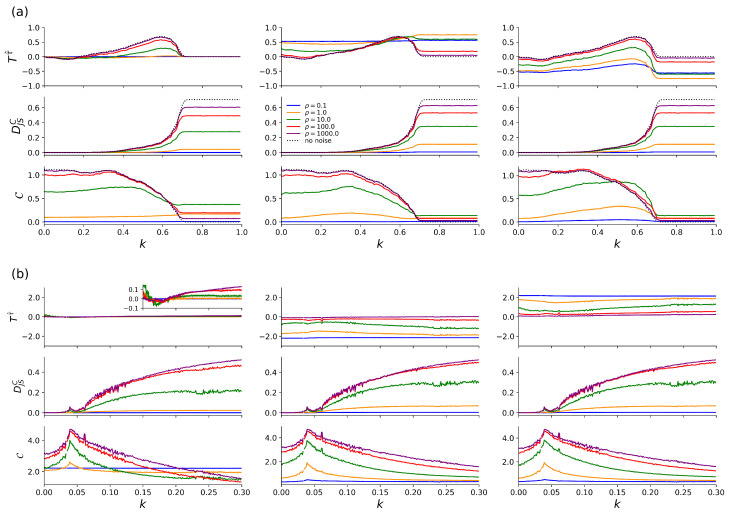
Dependence of transcript-based estimators for properties of interactions (directionality $T^{\left(\hat{\tau }\right)}$, strength $D_{\mathrm{JS}}^{C}$, complexity C) on the coupling strength *k* and under the influence of white noise with different signal-to-noise ratios ρ. Subfigures are ordered as follows: $f^{0}$-noise added to dynamics of both subsystems (left row), $f^{0}$-noise added to dynamics of first subsystem (middle row), $f^{0}$-noise added to dynamics of second subsystem (right row). (**a**): coupled Hénon maps; (**b**): coupled Rössler oscillators.

We first consider the noise-free case (see black dotted lines in Figure 1). For the coupled Hénon maps (Figure 1a), we expect the onset of complete synchronization at k=0.7 [43] at which the estimator for the strength of interaction $D_{\mathrm{JS}}^{C}$ attains its maximum value, while the estimator for the complexity of interaction C attains a minimum value. As expected for this driver-response configuration, the estimator for the direction of interaction $T^{\left(\hat{\tau }\right)}$ attains positive values only in some intermediate range of coupling strengths (0.2<k≲0.65), correctly indicating subsystem 1 driving subsystem 2. For the coupled Rössler oscillators (Figure 1b), we observe the onset of phase synchronization at k≈0.039 and fluctuations in the estimated strength of interaction $D_{\mathrm{JS}}^{C}$ for k∈[0.1,0.125] indicate intermittent lag synchronization [35,47]. In contrast to the coupled Hénon maps, complete synchronization is not observed for the investigated range of coupling strengths. The estimator for the complexity of interaction C decreases as the coupling strength increases and eventually vanishes for completely synchronized systems. As expected for this bidirectional coupling, the estimator for the direction of interaction $T^{\left(\hat{\tau }\right)}$ attains values close to 0 ($|T^{\left(\hat{\tau }\right)}|\leq 0.15$; cf. inset Figure 1b) for the whole range of coupling strengths.

Next, we consider the case of white-noise contamination that mimic measurement errors (Figure 1). For symmetric noise contamination ($f^{0}$-noise added to both subsystems), the values of all estimators for the properties of interactions decrease when the signal-to-noise ratio ρ decreases. If the noise equals or exceeds the signal in amplitude (ρ≤1), estimators lose their ability to correctly identify the direction of an interaction ($T^{\left(\hat{\tau }\right)}$; in case of unidirectional couplings) and distinguish coupling regimes and transitions to synchronization ($D_{\mathrm{JS}}^{C}$), as well as classify the complexity of an interaction (C). The latter may even be misclassified in the case of low signal-to-noise ratios, given the higher complexity of finite-length interacting stochastic processes (C≈2.2 for ρ=0.1).

Asymmetric noise contamination ($f^{0}$-noise added to one of the subsystems) have a quite strong impact on the estimator for the direction of interaction ($T^{\left(\hat{\tau }\right)}$). If noise is added to the driving subsystem of the coupled Hénon system, the values of $T^{\left(\hat{\tau }\right)}$ increase as the signal-to-noise ratio ρ decreases. Note, however, that the dependency on the coupling strength observed for the noise-free case and for symmetric noise contamination is lost with $T^{\left(\hat{\tau }\right)}$ converging to 0.5 for ρ=0.1. If noise is added to the responding subsystem, the values of $T^{\left(\hat{\tau }\right)}$ decrease as the signal-to-noise ratio ρ decreases, while the dependency on the coupling strength is largely preserved. $T^{\left(\hat{\tau }\right)}<0$ (for ρ≤1), however, indicates an inverted directionality, which makes the noisy responding system to appear as a driving system. Similar observations can be made for the bidirectionally coupled Rössler oscillators. When adding noise to the slightly faster subsystem 1, $T^{\left(\hat{\tau }\right)}$ attains more negative values as the signal-to-noise ratio decreases, indicating that subsystem 2 is a driving system. $T^{\left(\hat{\tau }\right)}$ attains more positive values if noise is added to the slightly slower subsystem 2, indicating that subsystem 1 is a driving system. Note that this de-/increase can already be observed for a signal-to-noise ratio of 100, which highlights the potential risk for misinterpretations. The values of the estimators for the strength and complexity of interactions decrease as the signal-to-noise ratio decreases. This observation holds irrespective of whether we add $f^{0}$-noise to the dynamics of either subsystem 1 or subsystem 2, and this is to be expected given the symmetry of these estimators under exchange of the subsystems.

Before we proceed with describing the influence of the other types of noise, let us introduce a measure for the deviation of the transcript-based estimators for properties of interactions from those of the noise-free case:

(20) $$\varepsilon_{A}=100\%\left[\frac{A_{\rho }-A_{0}}{\max|A_{0}|}\right],$$

where $A_{\rho }$ denotes the value of estimator $A\in \{T^{\left(\hat{\tau }\right)},D_{\mathrm{JS}}^{C},C\}$ for a signal-to-noise ratio of ρ, $A_{0}$ indicating the value of the noise-free case.

**Figure 2:**
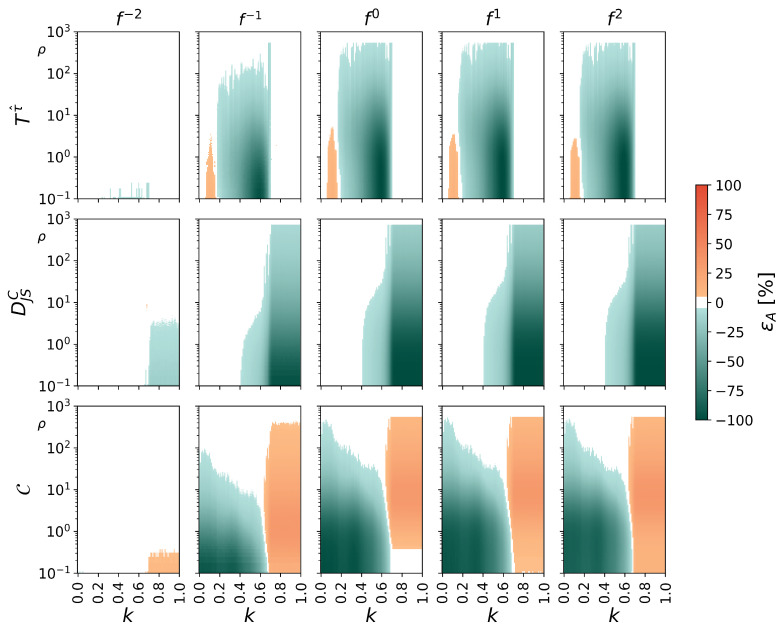
Percentage deviations $\varepsilon_{A}$ from the noise-free cases of transcript-based estimators for properties of interactions (top: directionality $A=T^{\left(\hat{\tau }\right)}$, middle: strength $A=D_{\mathrm{JS}}^{C}$, bottom: complexity A=C). Symmetric noise contamination of unidirectionally coupled Hénon maps, shown for different signal-to-noise ratios ρ and coupling strengths *k*. Deviations from the noise-free case not exceeding $|\varepsilon_{A}|=5\;\%$ are shown in white.

We start again with symmetric noise contamination of the unidirectionally coupled Hénon maps (Figure 2). A general observation can be made here, namely that contamination with $f^{-2}$-noise appear to have only a minor influence on transcript-based estimators for properties of interactions. If at all, they affect the estimators for strength and complexity of interactions for strong couplings (completely synchronized maps) and for comparably low signal-to-noise ratios (ρ≤1). The exact cause of this apparent robustness requires further investigation, but we conjecture that $f^{-2}$-noise hardly affects the fast dynamics of the Hénon map.

We observe that the estimator for the direction of interaction $T^{\left(\hat{\tau }\right)}$ exhibits the largest negative deviations from its noise-free values for intermediate coupling strengths k∈[0.2,0.7] and for almost all signal-to-noise ratios ρ ($\varepsilon_{T}^{\left(\hat{\tau }\right)}\geq 5\;\%$ for contamination with $f^{0}$-, $f^{1}$-, and $f^{2}$-noise already with ρ≈800, as well as for contamination with $f^{-1}$-noise with ρ≈200). Deviations decrease even further near the onset of complete synchronization at k=0.7, and they vanish for k>0.7, where there is no flow of information (cf. Figure 1). For small couplings (k<0.2), deviations increase for decreasing ρ.

For $D_{\mathrm{JS}}^{C}$, the estimator for the strength of interactions, we observe the largest deviations for strong couplings (k≥0.7), where the noise-free maps are already completely synchronized (cf. Figure 1). Deviations can be observed for all types of noise and all signal-to-noise ratios, although less pronounced for contamination with $f^{-2}$-noise. As $D_{\mathrm{JS}}^{C}$ depends sensitively on the distributions of ordinal patterns in the source and target time series and attains its maximum value only for uniformly distributed transcripts all belonging to order class 1, even tiny deviations from these distributions can lead to noticeable underestimations of the coupling intensity and thus to erroneous classifications of the synchronized dynamics. For intermediate couplings (0.4<k<0.7), where $D_{\mathrm{JS}}^{C}$ indicates the transition to complete synchronization in the noise-free case, we observe significant deviations for contamination with almost all types of noise (once again, $f^{-2}$-noise is an exception) already at high signal-to-noise ratios (ρ≲400). The apparent noise robustness for k<0.4 can be explained by the comparably low sensitivity of $D_{\mathrm{JS}}^{C}$ for small couplings (cf. Figure 1).

Complexity of an interaction, estimated with C, appears to be least robust against noise contamination. It exhibits deviations from the noise-free values for almost all couplings and almost all investigated signal-to-noise ratios (exceptions can be observed for weak-to-intermediate couplings at high signal-to-noise ratios (ρ>100, on average). For strong couplings (k>0.7), the interaction contains no information and noise contamination lead to increased values of C, given that noise-induced fluctuations in the ordinal patterns of the source and target lead to an increase in perceived complexity. For weak-to-intermediate couplings at larger signal-to-noise ratios (ρ≲100), C decreases until we are left with essentially two realizations of noise, whose relationship also yields no information.

**Figure 3:**
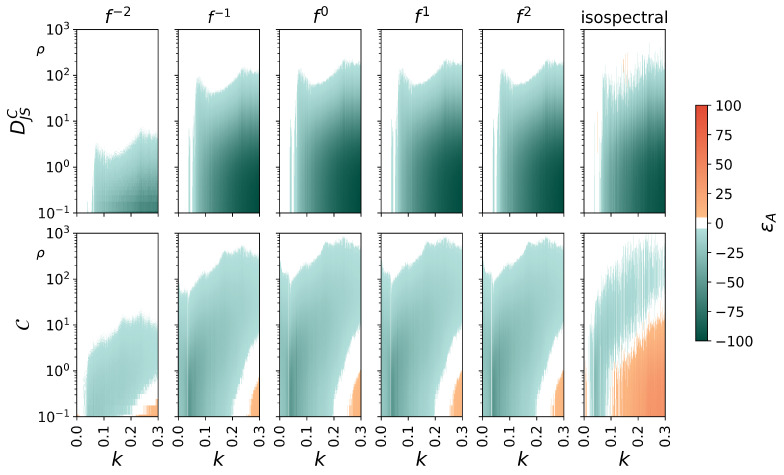
Percentage deviations $\varepsilon_{A}$ from the noise-free cases of transcript-based estimators for properties of interactions (top: strength $D_{\mathrm{JS}}^{C}$, bottom: complexity C) for symmetric noise contamination, including contamination with isospectral noise, of bidirectionally coupled Rössler oscillators relative to the noise-free cases, shown for different signal-to-noise ratios ρ and coupling strengths *k*. Deviations from the noise-free case not exceeding $|\varepsilon_{A}|=5\;\%$ are shown in white.

Figure 3 summarizes our findings on symmetric noise contamination of the bidirectionally coupled Rössler oscillators. Since deviations of the estimator for the direction of interaction $T^{\left(\hat{\tau }\right)}$ from the values for the noise-free cases ($T^{\left(\hat{\tau }\right)}\approx 0$) were negligible for all types of noise, we refrain from presenting these results.

The estimator for the strength of interactions $D_{\mathrm{JS}}^{C}$ seems to be quite susceptible to symmetric noise contamination, regardless of the spectral content of the noise. Already for comparably high signal-to-noise ratios (20≲ρ≲100), the coupling intensity is consistently underestimated within most of the investigated range of couplings strengths *k*, and deviations increase with decreasing ρ.

We make similar observations for the estimator of the complexity of interaction C. Furthermore, complexity of interaction is overestimated for higher couplings (k≳0.25) in the presence of high-amplitude noise (ρ<1). Note that such an overestimation is even more pronounced in the presence of isospectral noise: it can be observed for even smaller couplings (k≲0.1) and for higher signal-to-noise ratios (0.1≤ρ≤10).

**Figure A1:**
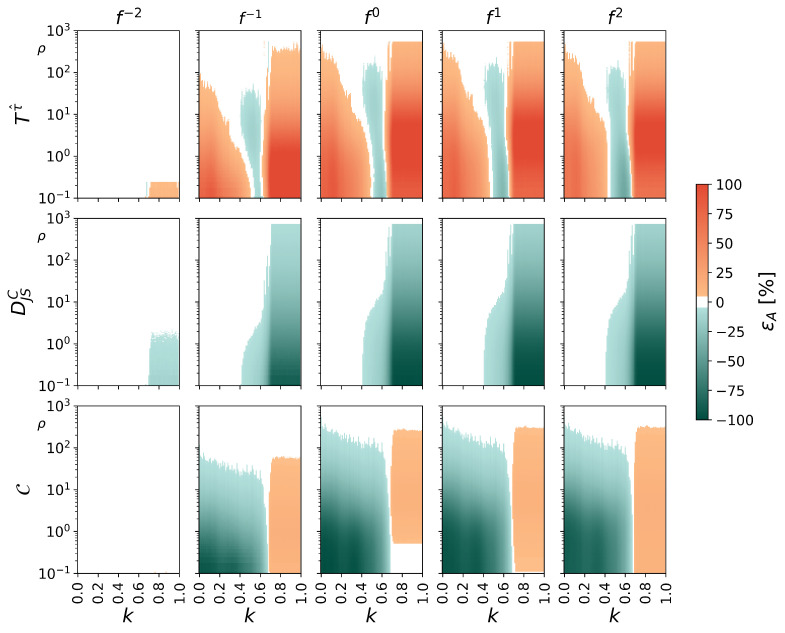
Same as Figure 2 in main text, but for noise added to dynamics of subsystem 1 (driver) of the unidirectionally coupled Hénon maps.

**Figure A2:**
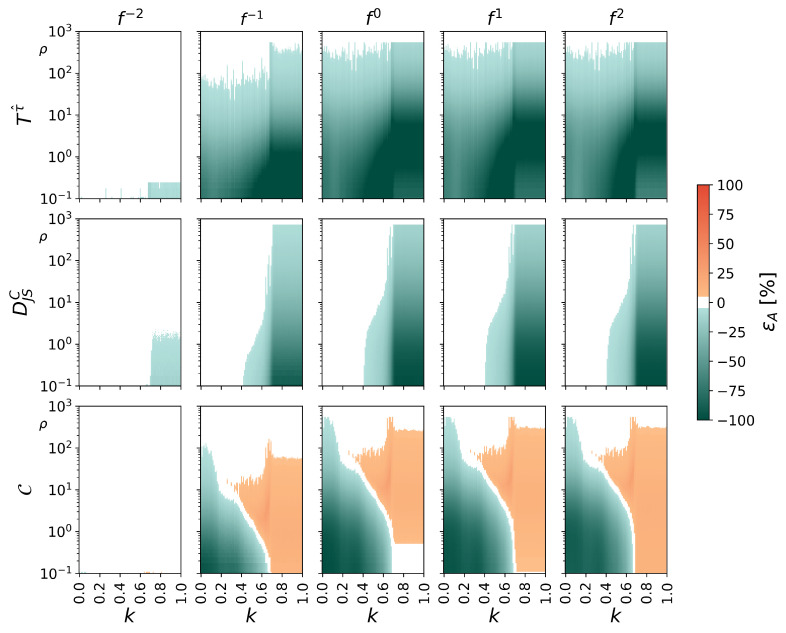
Same as Figure A1, but for noise added to dynamics of subsystem 2 (response).

**Figure A3:**
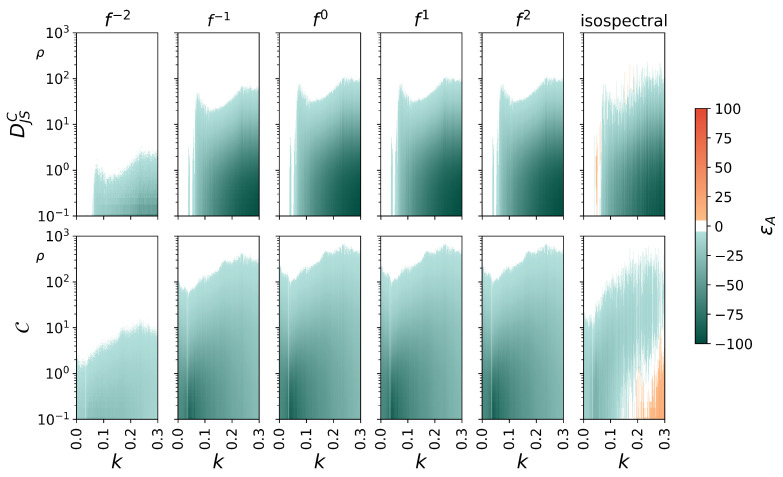
Same as Figure 3 in main text, but for noise, including isospectral noise, added to dynamics of subsystem 1 (faster oscillator) of the bidirectionally coupled Rössler oscillators. We again refrain from presenting the negligible effects on the estimator for the direction of interaction $T^{\left(\hat{\tau }\right)}$.

**Figure A4:**
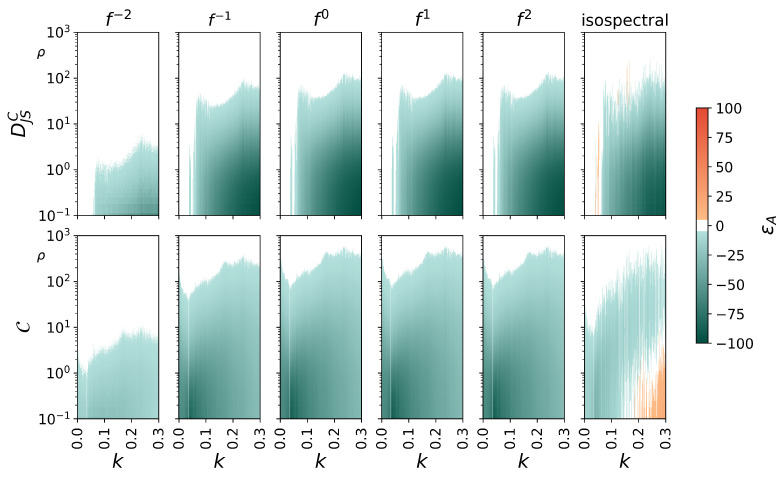
Same as Figure A3, but with noise added to dynamics of subsystem 2 (slower oscillator) of the coupled Rössler oscillators.

We also report our findings for asymmetric noise contamination (noise added to the dynamics of subsystem 1 or subsystem 2), restricting ourselves to the impact of the different types of noise on the estimator for the direction of interaction $T^{\left(\hat{\tau }\right)}$ at two exemplary coupling strengths. A presentation of the full results can be found in Appendix A (Figure A1 and Figure A2 for the coupled Hénon maps; Figure A3 and Figure A4 for the coupled Rössler oscillators).

**Figure 4:**
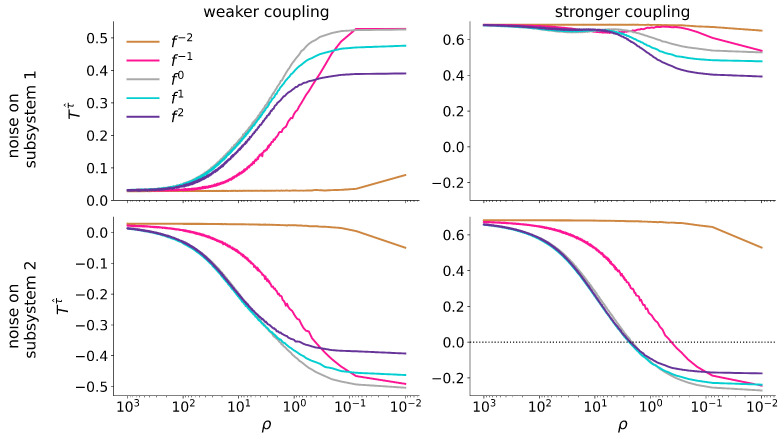
Estimator for the direction of interaction $T^{\left(\hat{\tau }\right)}$ between unidirectionally coupled Hénon maps (left column: weaker coupling (k=0.2); right column: stronger coupling (k=0.6)) for different signal-to-noise ratios ρ. Colors encode different type of noise (see legend). Top row: noise added to dynamics of subsystem 1 (here: driver), bottom row: noise added to dynamics of subsystem 2 (here: response).

Figure 4 shows—for all types of noise—how $T^{\left(\hat{\tau }\right)}$ between the unidirectionally coupled Hénon maps varies with the signal-to-noise ratio ρ (we again note the only minor impact of $f^{-2}$-noise). For weakly coupled maps, $T^{\left(\hat{\tau }\right)}$ attains increasingly positive (negative) values with higher amplitudes of the noise added to subsystem 1 (subsystem 2), eventually converging to some constant value that differs for the different types of noise. In cases where the actual coupling strength *k* is not known, the attained values would correctly indicate the driver and response subsystems. However, this indication must be considered spurious as we expect $T^{\left(\hat{\tau }\right)}\approx 0$ for this weak coupling. For more strongly coupled maps, adding noise to subsystem 1 does not alter its identification as the driver subsystem, neither for the different types of noise nor for the range of signal-to-noise ratios considered here. In contrast, adding noise to subsystem 2 (response) can lead to falsely identifying this subsystem as a driver. For $f^{0}$-, $f^{1}$-, and $f^{2}$-noise, we observe instances of misidentification for ρ≈2 and slightly higher noise amplitudes (ρ≲0.3) suffice for contamination with $f^{-1}$-noise.

**Figure 5:**
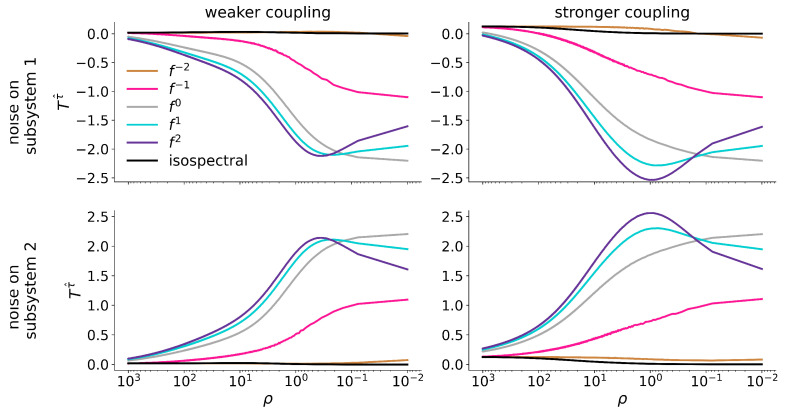
Same as Figure 4, but for the bidirectionally coupled Rössler oscillators (left column: weaker coupling (k=0.07); right column: stronger coupling (k=0.25). Top row: noise added to dynamics of subsystem 1 (here: faster oscillator), bottom row: noise added to dynamics of subsystem 2 (here: slower oscillator).

**Figure A5:**
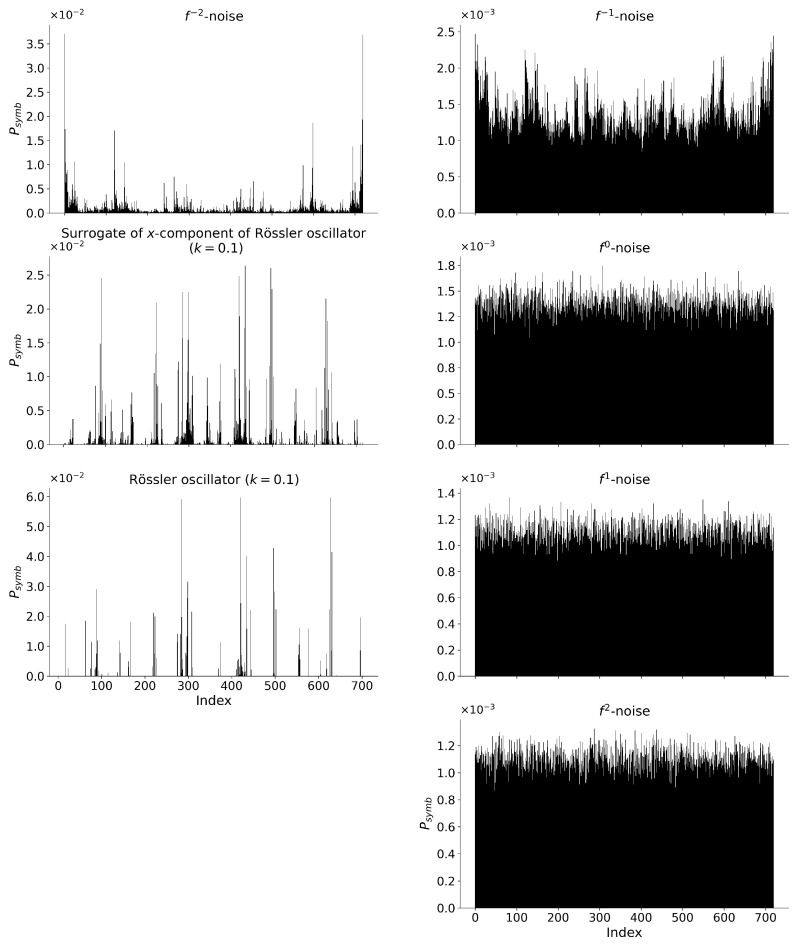
Frequency distributions of ordinal patterns derived from different time series. x-axis shows the lexicographic index of an ordinal pattern.

For the coupled Rössler oscillators (Figure 5), we again observe that adding noise to the dynamics of subsystem 1 (2) leads to more negative (positive) values of $T^{\left(\hat{\tau }\right)}$ which indicates subsystem 2 (1) to be a driving system. This holds for both coupling strengths and for almost the full range of signal-to-noise ratios (note that for contamination with $f^{2}$- and $f^{1}$-noise the increase/decrease is not monotonous but rather surpasses a global maximum/minimum, reminiscent of stochastic resonance [48]). Interestingly, the impact of $f^{-2}$- and isospectral noise appears to be negligible for this system, and we conjecture that the apparent noise robustness may be due to comparable density distributions of ordinal patterns of these noise types and of the system (cf. Figure A5 in Appendix A).

## 5. Discussion

Ordinal pattern-based estimators for properties of dynamical systems and for interactions between them are often considered as comparably robust against the influence of measurement noise. In real-world applications (see, e.g., [49] and references therein), however, one encounters contamination with other types of noise, ranging from colored noise to isospectral noise, and the contamination might be symmetric (comparable noise in all subsystems) or asymmetric (noise differs in all subsystems).

Using paradigmatic coupled model systems (unidirectionally coupled Hénon maps and bidirectionally coupled Rössler oscillators), we here investigated the robustness of transcript-based estimators for properties of interactions [16,35,36,37] against symmetric and asymmetric contamination with different types of noise. We observed that all estimators exhibited noticeable deviations from their noise-free values already for signal-to-noise ratios orders of magnitude higher than the usual ratios one typically encounters in real-world applications. This holds true, regardless of the type of noise and for both symmetric and asymmetric contamination. This dependence might have a comparably lower impact on estimators for strength and complexity of interaction, as different coupling regimes can still be distinguished and characterized sufficiently well. In contrast, for the estimator for the direction of interaction this dependence bears the potential risk for misinterpretations (the more noisy, more active, or faster subsystem is falsely identified as driver), in line with the common notion that the inference of causality—particularly from short and noisy time series—is notoriously problematic [22,49,50,51,52,53,54,55]. The use of specially designed surrogate tests (e.g., [56,57]) and further advances in ordinal pattern analyses [58,59] could lead to improvements.

With our studies, we observed some dependencies on contamination with $f^{-2}$-noise that clearly differed from those seen with other types of noise. We could not yet pinpoint the exact origin(s) of these dependencies, but we are confident that further investigations will provide more insights.

## Data Availability

No new data were created or analyzed in this study. Data sharing is not applicable to this article.
